# Supplementary material for: Molecular insights into biogenesis of glycosylphosphatidylinositol anchor proteins
Source: Nat Commun. 2022 May 12;13:2617. doi: 10.1038/s41467-022-30250-6 (PMC9098846; doi:10.1038/s41467-022-30250-6)
Supplement: Supplementary file 1 — Supplementary Information [file 41467_2022_30250_MOESM1_ESM.pdf]

Title: Molecular insights into biogenesis of glycosylphosphatidylinositol anchor proteins

Authors: Yidan Xu[1, †], Guowen Jia[2, †], Tingting Li[1, †], Zixuan Zhou[3, †], Yitian Luo[1], Yulin Chao[3], Juan Bao[1], Zhaoming Su[2, *], Qianhui Qu[3, *], Dianfan Li[1, *]

Supplementary Figures 1-12

Supplementary Table 1

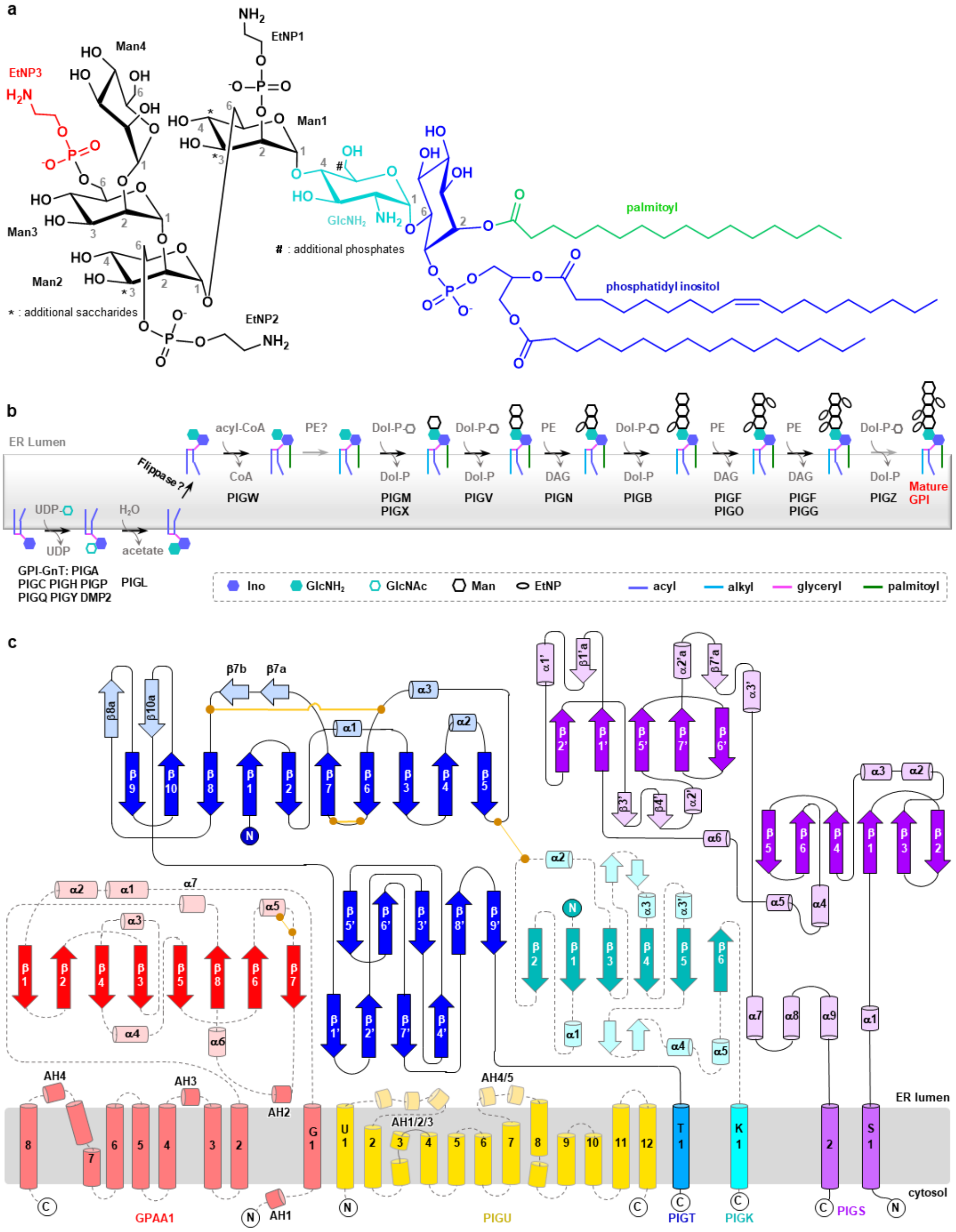

Supplementary Fig. 1. Chemical structure of GPI and topology of GPI-T. a The GPI core contains a phosphatidyl inositol (blue), a palmitoyl chain (green), a glucosylamine ($GlcNH_2$) (cyan), and four mannoses (Man) with possible modification of ethanolamine phosphate (EtNP) on Man1/2. The reactive EtNP3 on Man3 is colored red. Relevant carbon atoms on the sugar ring are numbered. The glycan core can be further decorated with saccharides (asterisk) or phosphates (hash). The fatty chain on the *sn*-1 position of the glycerol backbone is drawn as an acyl group but it can be an alkyl group. **b** Biosynthesis of GPI. Substrates and by-products of each reaction are labeled with grey

text. Enzymes catalyzing the reactions are indicated with black texts. The two steps that do not always occur are indicated with a grey arrow. A question marker denotes speculative participants. The mature GPI is used by GPI-T. Enzymatic steps carrying further modifications are not shown. The shaded box indicates the endoplasmic reticulum (ER) membrane. Various components are explained in the dashed box. The pathway cartoon was redrawn based on ref. [1]. **c** Topology of GPI-T drawn with structural knowledge from this study. Subunits are colored differently (red, GPAA1; orange, PIGU; blue, PIGT; cyan, PIGK; purple, PIGS). The soluble regions are colored darker than the membrane-associated regions. Connected orange dots indicate disulfide bonds. Transmembrane helices (TMHs) are indicated by numbers and the ER membrane is shaded grey. Major secondary structures are numbered to match the description in the main text and Supplementary Fig. 2. Loops connecting major secondary elements are shown as dashed lines (GPAA1, PIGU, PIGK) or solid lines (PIGT, PIGS). AH, amphipathic helix. N/C indicates the N- and C-terminal of subunits. Abbreviations: CoA, coenzyme A; DAG, diacylglycerol; Dol-P, dolichol phosphate; EtNP, ethanolamine phosphate; GlcNAc, N-acetylglucosamine; $GlcNH_2$, glucosamine; Ino, inositol; Man, mannose; PE, phosphatidylethanolamine; UDP, uridine diphosphate.

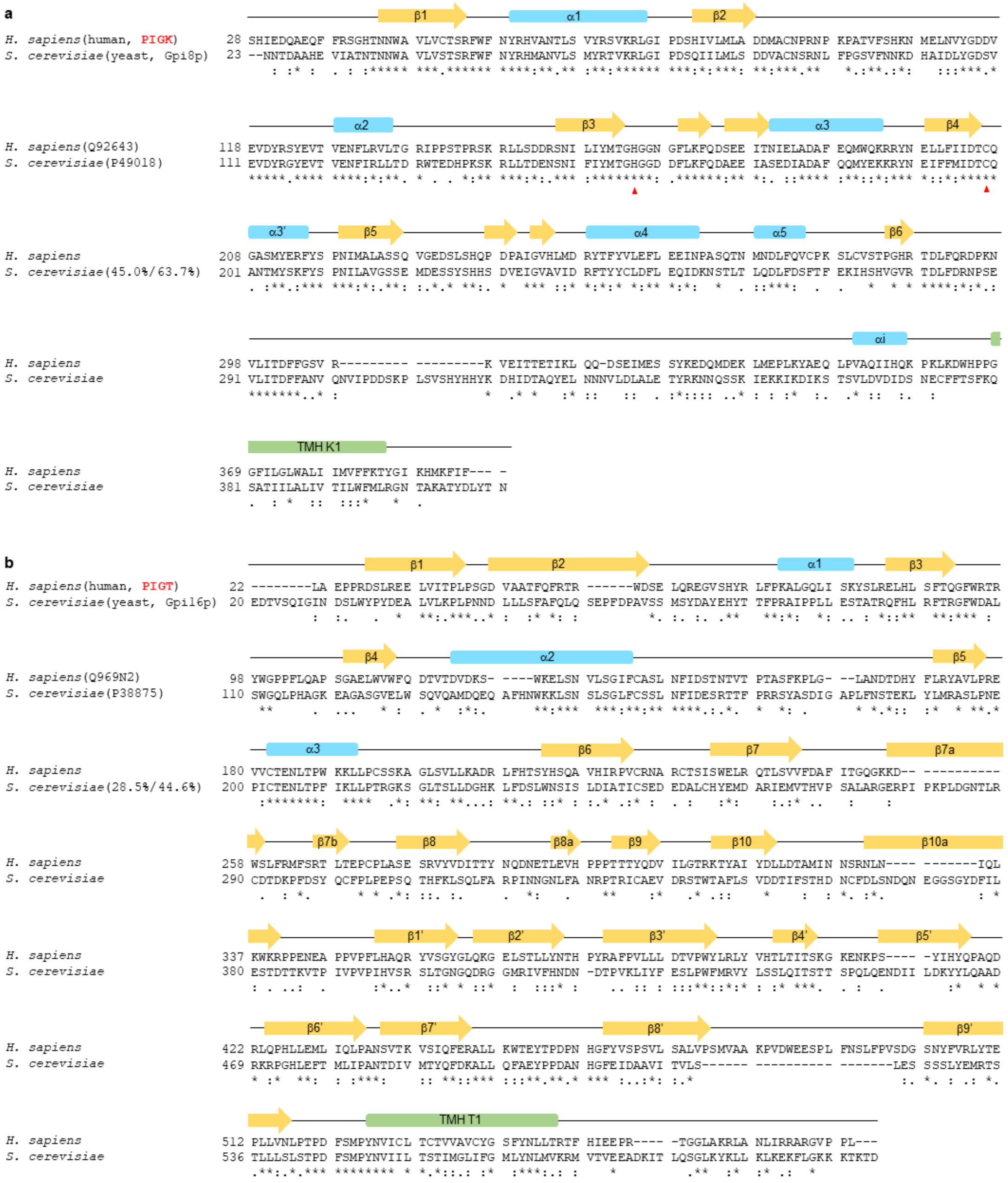

To be continued onto the next page

Continued from the previous page

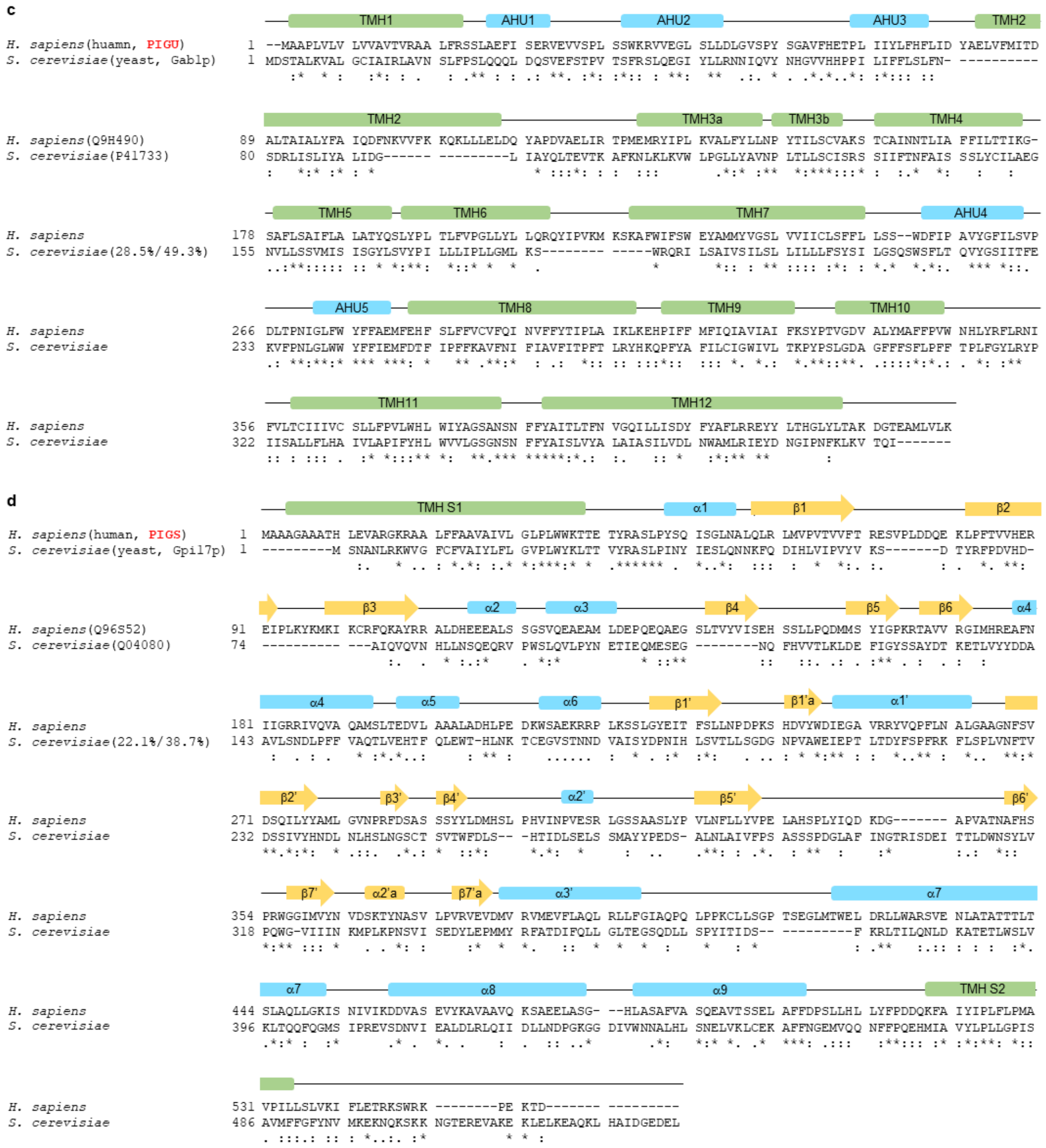

To be continued onto the next page

Continued from the previous page

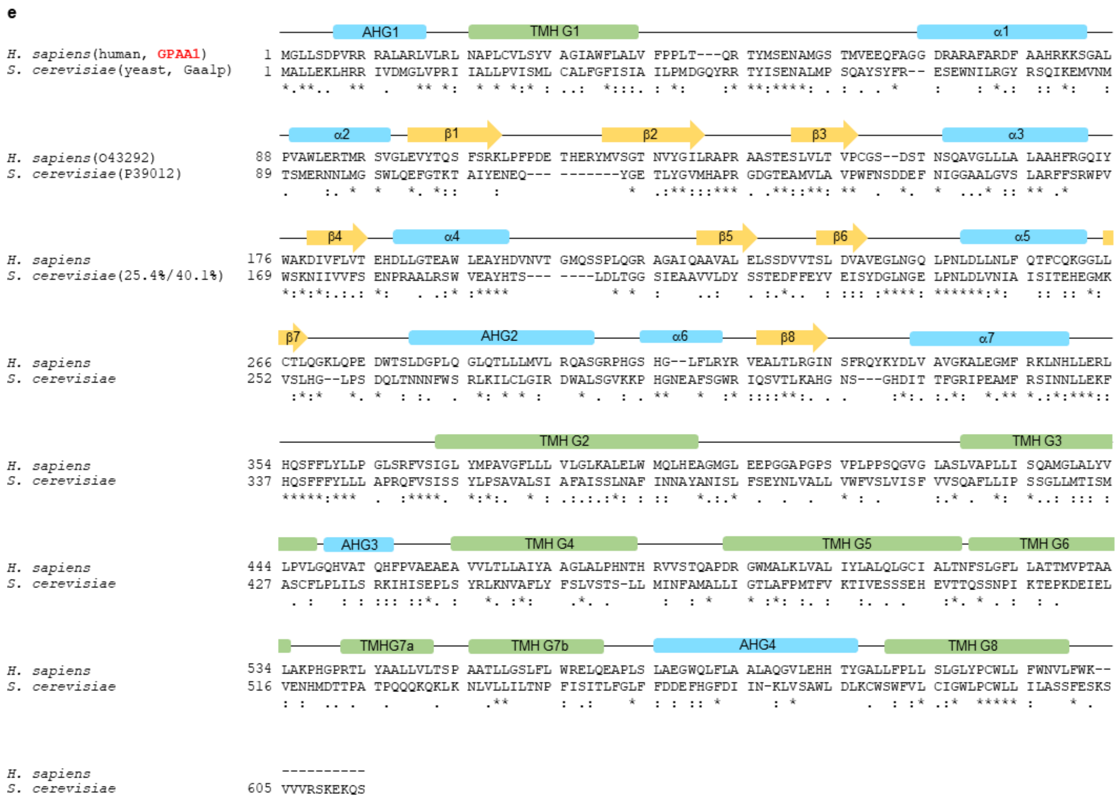

Supplementary Fig. 2. Sequence alignment of GPI-T from human (*Homo sapiens*) and yeast (*Saccharomyces cerevisiae*). a PIGK. The catalytic dyad residues are marked with a triangle. **b** PIGT; **c** PIGU; **d** PIGS; **e** GPAA1. Uniprot IDs are included along with the sequence identity/similarity compared to the human orthologues. Secondary structure elements of the human GPI-T are labeled based on the 3D structure determined in this work. Asterisk, colon, and dot indicate identical, conserved, and semi-conserved substitutions, respectively. Secondary structure segments are number sequentially unless they are called differently in the text for ease of discussions in some cases such as structure comparison and for sub-domain descriptions. Transmembrane helices (TMHs) and amphipathic helices (AHs) are labeled with a letter referring to different subunits (G, GPAA1; K, PIGK; S, PIGS; T, PIGT; U, PIGU) followed by a number. Major secondary structural elements are labeled with the type (α, α-helix; β, β-strand) followed by a number. A prime symbol in PIGK marks additional elements compared with legumains/caspases (Supplementary Fig. 4). A prime symbol in PIGT/PIGS differentiates the two different domains. In PIGT, a letter ("a"/"b") at the end marks β-strands that are not a part of the main β-sheet (Fig. 4b). In PIGU/GPAA1, a letter ("a"/"b") indicates the breaking of TMHs. In PIGS, a letter ("a") indicates additional β-strands compared to its structural homolog metazincin (Supplementary Fig. 7).

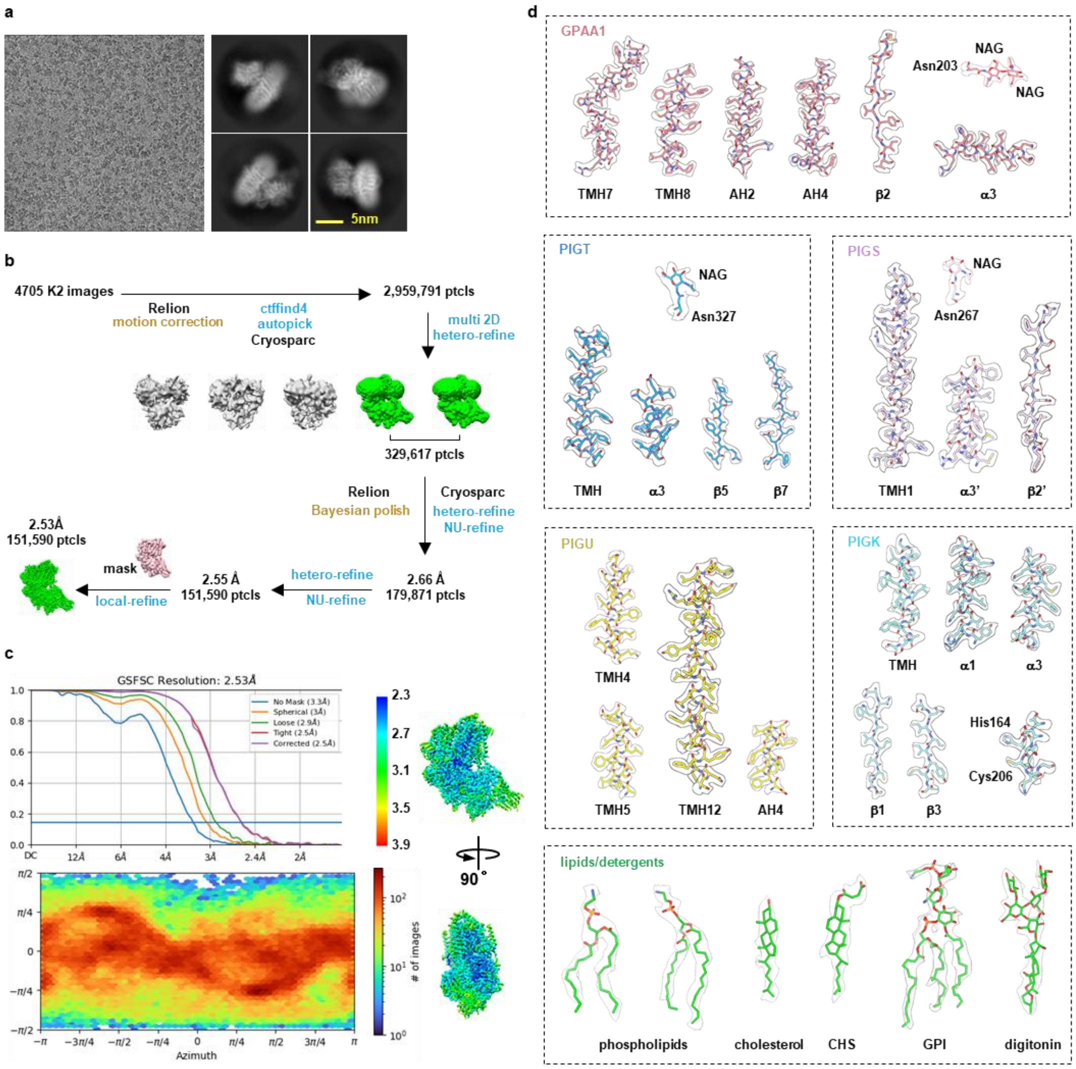

Supplementary Fig. 3. Cryo-EM data processing and high-quality density/model fitting exemplary views. a Representative cryo-EM micrograph of GPI-T and selected 2D class averages. **b** The workflow of classification and refinement. **c** The nominal resolution of GPI-T was determined by the 'gold-standard' Fourier shell correlation (FSC) curve using the FSC=0.143 criterion, and angular distribution heatmap at the bottom panel calculated in Cryosparc. Local resolution evaluation was shown on right. **d** Cryo-EM map density and model of representative protein parts, glycosylation sites, and lipids and detergents. AH, amphipathic helix; CHS, cholesteryl hemisuccinate; GPI, glycophosphatidylinositol; NAG, N-acetyl glucosamine; ptcls, particles; TMH, transmembrane helix.

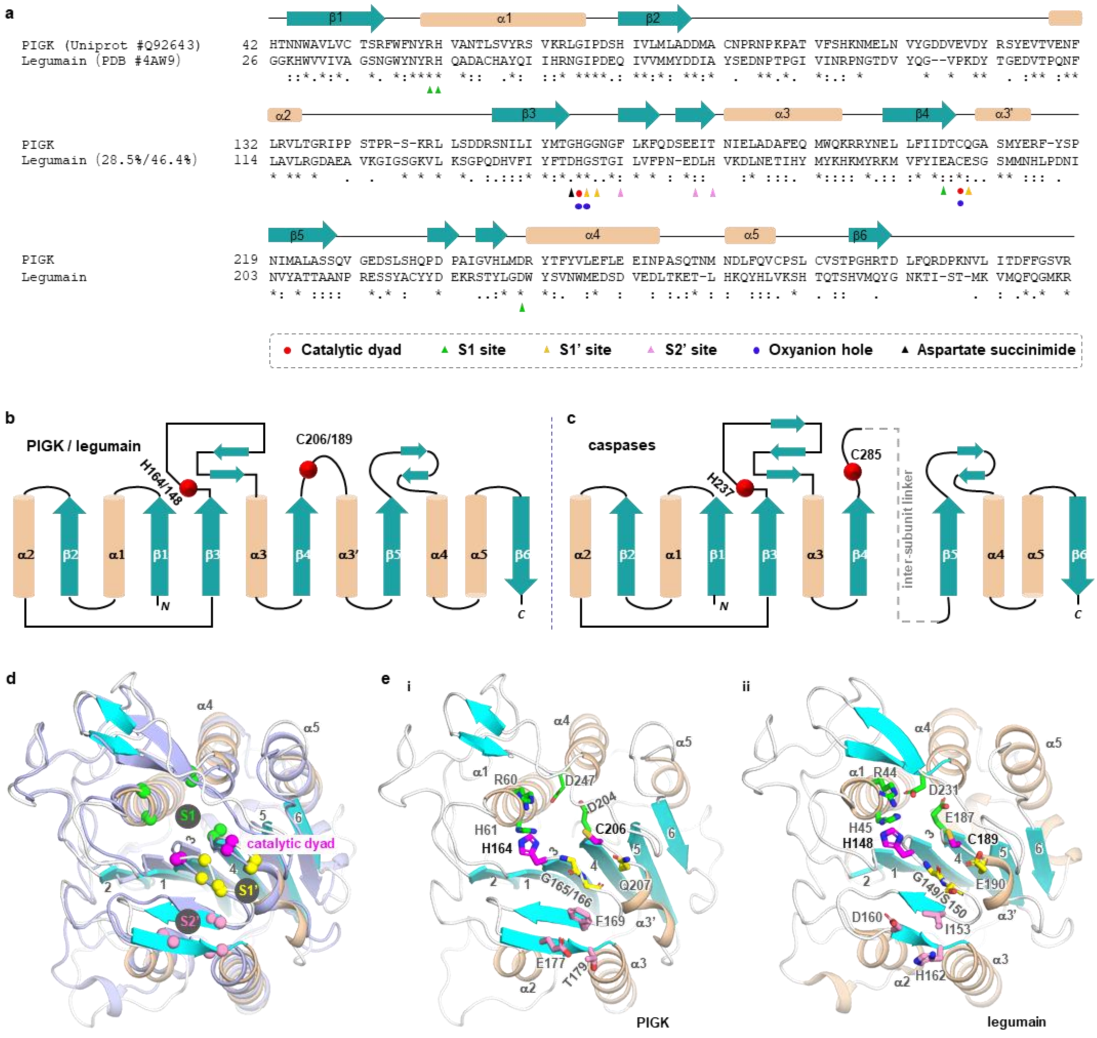

Supplementary Fig. 4. PIGK shares sequence and structure similarity with legumains and caspases. a Sequence alignment between PIGK and legumain. Asterisk, colon, and dot indicate identical, conserved, and semi-conserved substitutions, respectively. Labels of elements relating to substrate-binding and catalysis are explained in the dashed box. Uniprot ID and sequence identity/similarity are indicated appropriately. Major secondary structural elements are labeled sequentially with the type (α, α-helix; β, β-strand) followed by a number. A prime symbol marks additional elements compared with legumains/caspases**. b, c** PIGK shares a similar topology with legumains [2] (**b**) and caspases [3] (**c**). The linker region that was removed in the mature caspases is shown as a dashed line. **d, e** High structural similarity (*Z*-score of 29.6 and Cα-RMSD of 2.3 Å by the DALI server) [4] between PIGK and legumains as revealed by superposition (**d**, PIGK, cyan/wheat for β-strands/α-helices; legumain, blue, PDB ID 4AWB [http://doi.org/10.2210/pdb4AWB/pdb]) and side-by-side comparison (**e**). The six β-strands (1-6) in the central sheet and connecting a-helices (α1- α6) are labeled. The catalytic dyad and S1/S1'/S2' residues are shown as Cα spheres (**d**) or sticks (**e**) and colored magenta, green/yellow/pink, respectively.

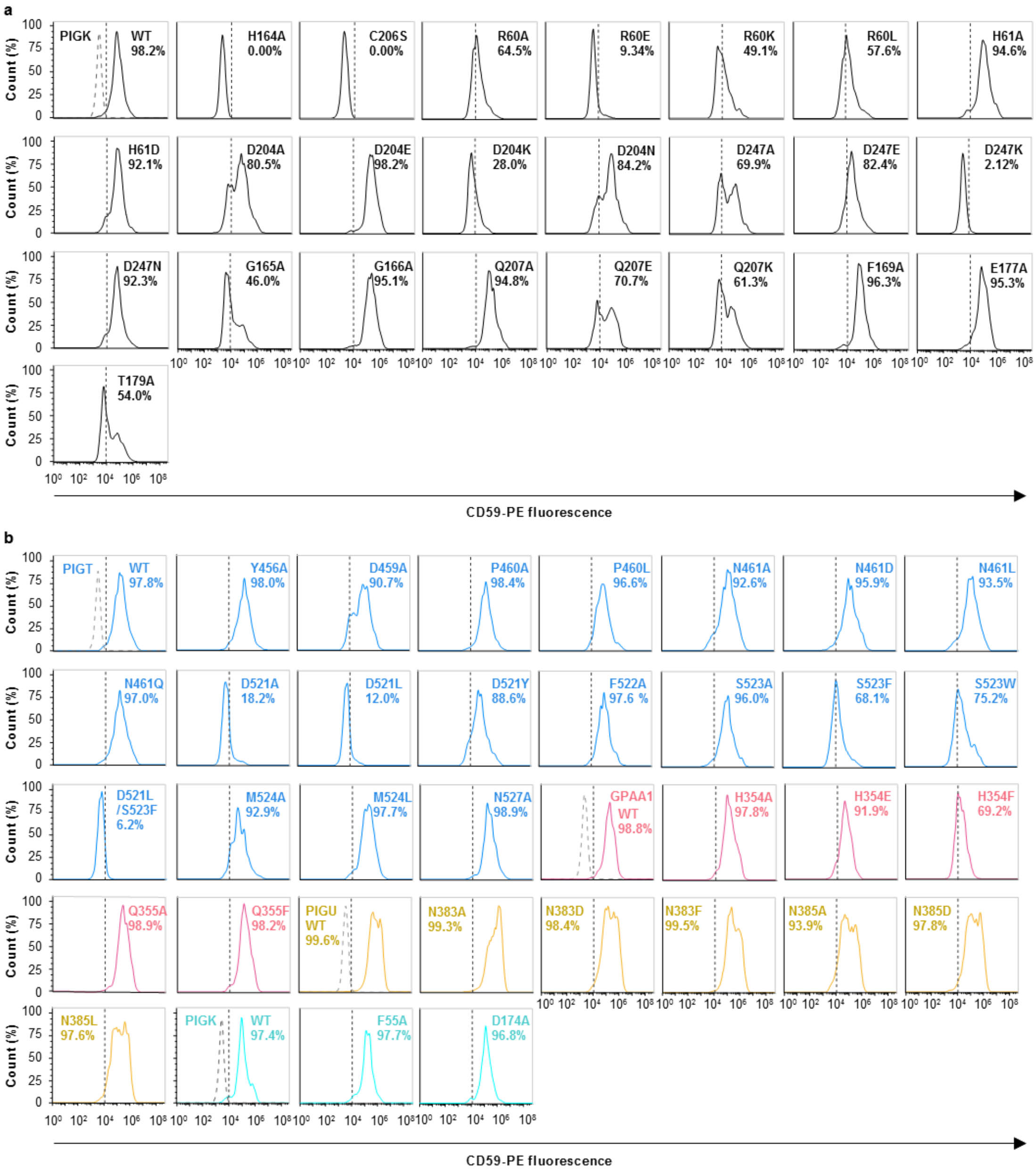

Supplementary Fig. 5. Typical fluorescence-activated cell sorting (FACS) results for the constructs described in the main text. a Representative FACS results of three independent experiments for the PIGK mutants (the catalytic dyad and S1/S1'/S2' sites). **b** Representative FACS results of three independent experiments for the mutants of the GPI-binding site residues. The percentage shown in this figure represents the subpopulation of CD59-positive cells (as the function of GPI-AP processing) in the TGP-positive population (as a result of subunit expression). To calculate activity, the percentage shown in this figure was normalized using a negative control (grey dashed line and the vertical dash line, cells expressing an unrelated TGP-tagged membrane protein) and a positive control (cells expressing the wild-type). The expression and integrity of the subunits were also separately verified by SDS-PAGE in-gel fluorescence (Supplementary Fig. 10). Results are grouped by subunits and the traces are color-coded by subunits (blue, PIGT; red, GPAA1; orange, PIGU; cyan, PIGK). PE, phycoerythrin.

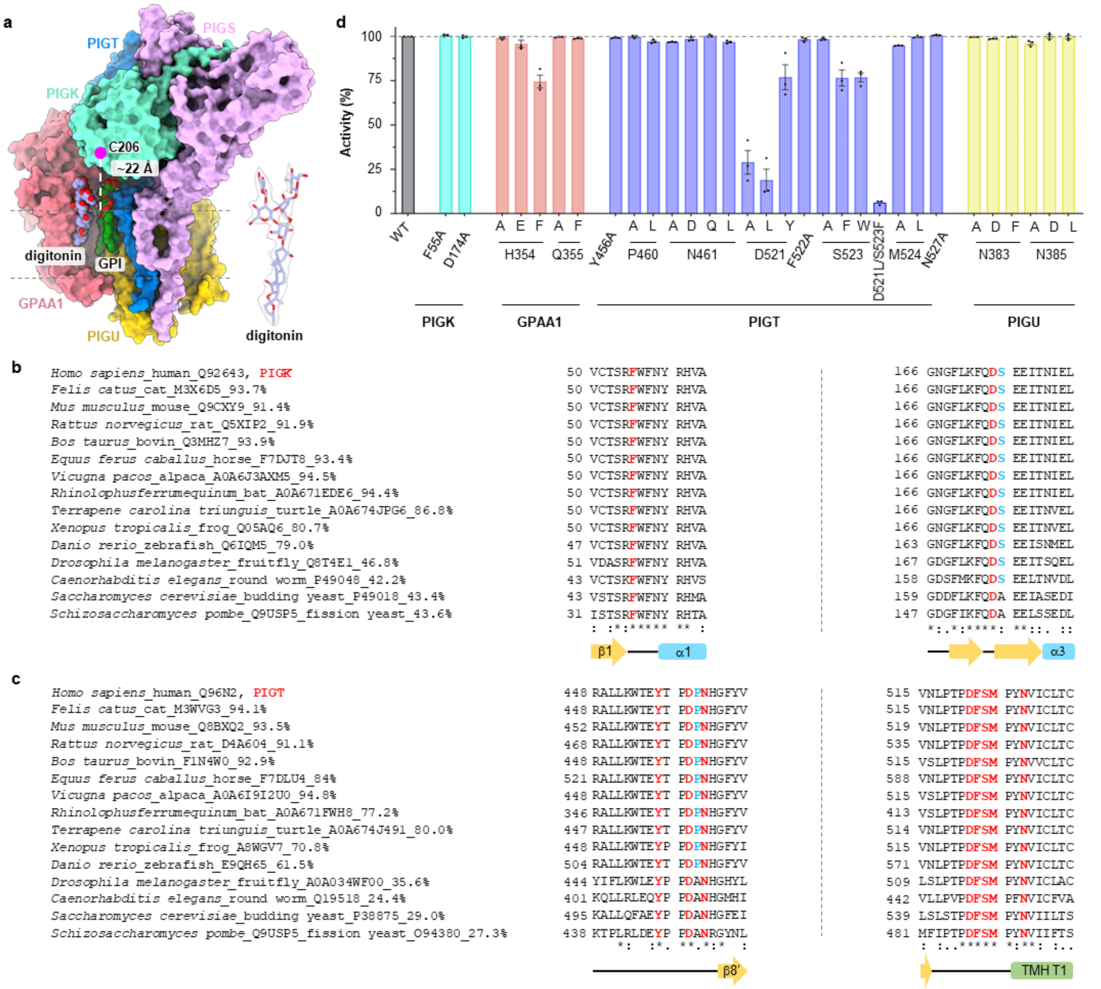

Supplementary Fig. 6. The GPI-binding site is delineated with conserved residues and hosts a digitonin molecule. a A digitonin (light blue) which also contains a polyglycan chain is found near GPI (green). The cryo-EM density of the digitonin is shown in the right corner. The GPI-T structure is shown as surface representation color-coded by subunits as indicated. The membrane boundary is shown by the dashed lines. **b, c** Sequence alignment for segments of PIGK (**b**) and PIGT (**c**) (red, completely conserved; cyan, highly conserved) around the GPI-binding site (Fig. 3b). Protein sequences were selected from evolutionarily representative species. Uniprot IDs of the PIGT/PIGK sequences and their sequence identity to the human ortholog are shown along with the binomial nomenclature and common names. Asterisk, colon, and dot indicate identical, conserved, and semi-conserved substitutions, respectively. Secondary elements were labeled for easier location of the corresponding elements in Supplementary Fig. 2. **d** Apparent activity of GPI-binding site mutants relative to the wildtype (WT). Activity was measured by immune staining of a reporter GPI-AP (CD59) on the surface of knockout cells transfected with the indicated mutants. Data represent mean ± s.e.m. from three independent experiments. Activity for PIGT D521/S523, GPAA1 H354, and PIGU N385 are re-plotted using data from Fig. 3d. The bar graph is color-coded to match the coloring of subunits in **a**. Source data are provided.

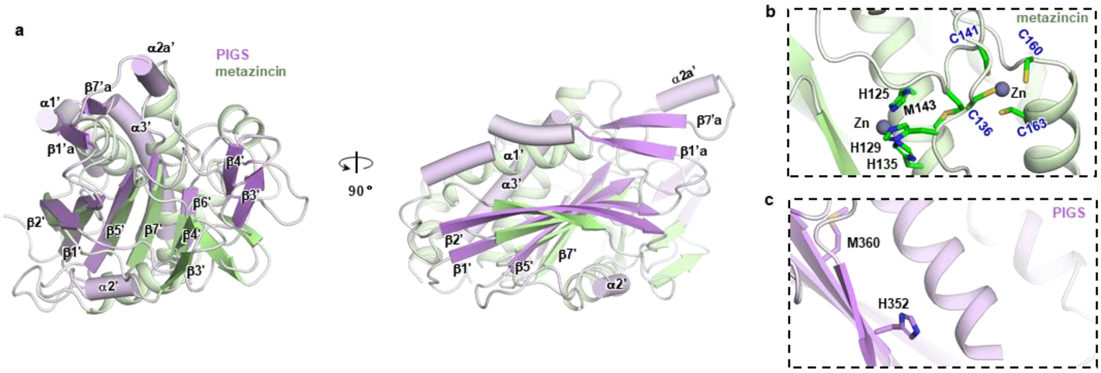

Supplementary Fig. 7. PIGS contains a protease-like domain. a The PIGS domain containing residues 222-398 (purple, cylinder) shares structural similarity with the metazincin AmzA (green, cartoon, PDB ID 2X7M [http://doi.org/10.2210/pdb2X7M/pdb]) with a Z-score of 5.9 and Cα RMSD of 3.6 Å) [4]. Major secondary structures are numbered to match the description in the main text and Supplementary Fig. 2d. **b, c** The two Zn-binding motifs in metazincin (**b**) are compositionally impossible for PIGS (**c**) as only one histidine and one methionine were found in PIGS in the vicinity (within 10 Å).

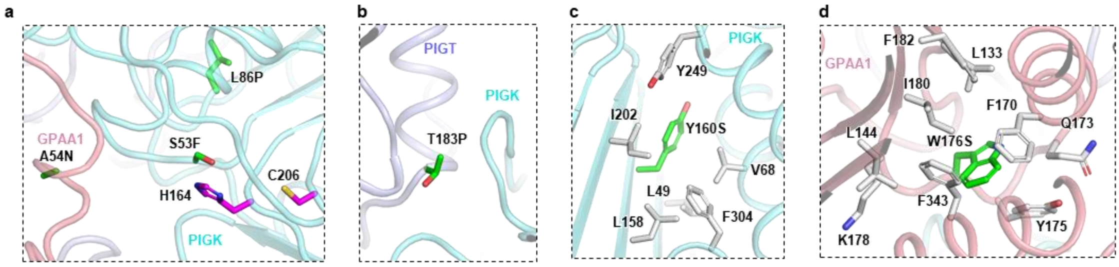

Supplementary Fig. 8. Expanded view of the distribution of representative genetic mutations. a, b Exemplary expanded view of residues close to the catalytic dyad (**a**) and at the inter-subunit interfaces (**b**). **c, d** The PIGK Y160S (**c**) and PIGT W176S (**d**) may cause folding issues by introducing a small hydrophilic residue into a large hydrophobic pocket. Portions of subunits are shown as ribbon representations color-coded as indicated. Amino acid residues of interest are shown as stick representations, with those bearing disease mutations colored green, the catalytic dyad residues colored magenta, and the one in the local environments colored grey.

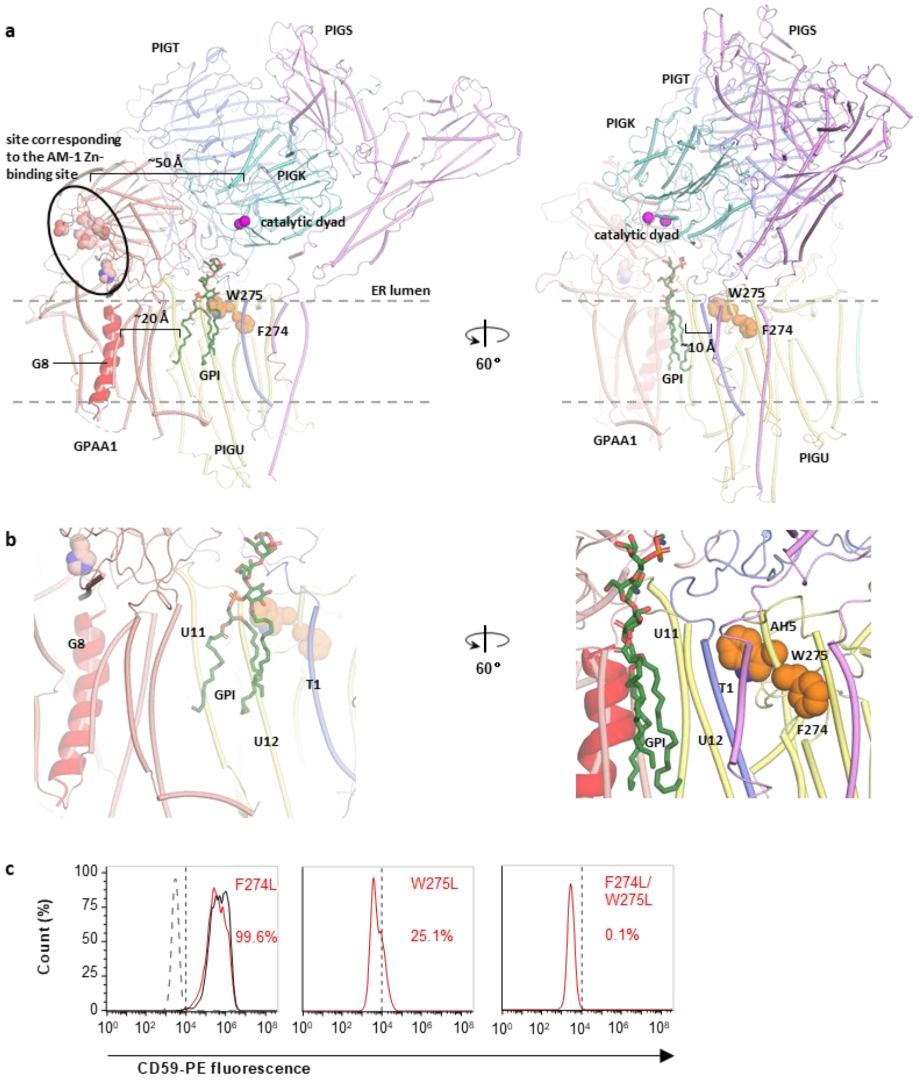

Supplementary Fig. 9. The site corresponding to the AM-1 Zn-binding site, the TMH8 of GPAA1, and F274/W275 in PIGU are not at the proposed active site. a Overview. **b** Expanded view. Subunits and various components are colored as indicated and labeled appropriately. The catalytic dyad is shown as Cα spheres (magenta). F274/W275 are shown as sphere representations (orange). Relevant transmembrane helices are labeled with a letter referring to the subunits (G, GPAA1; T, PIGT; U, PIGU) and a number referring to the number of the helix. **c** Functional importance of PIGU F274/W275. The function of wild-type PIGU (black) and mutants (red) were assessed by monitoring the surface expression of CD59 in PIGU knockout cells. Cells were gated by TGP fluorescence [5] for GPAA1 expression and analyzed for CD59 staining using phycoerythrin (PE)-conjugating antibodies. The dotted line (grey) indicates background staining of cells expressing an unrelated TGP-tagged membrane protein (negative control). A vertical dash line marks the threshold (CD59-gating) determined from the negative control. Shown is a representative result of three independent experiments. Data for all the three experiments are included in the Source Data File.

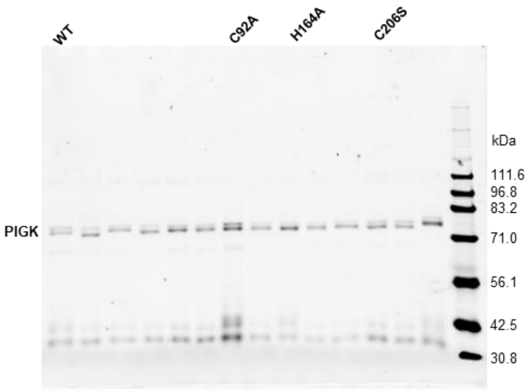

Related to Fig. 2b

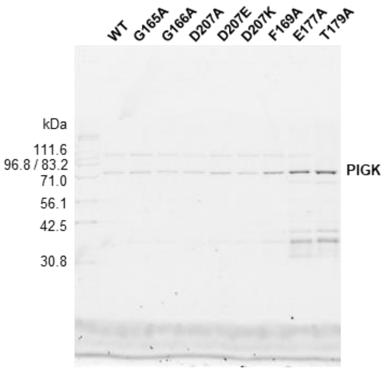

Related to Fig. 2b

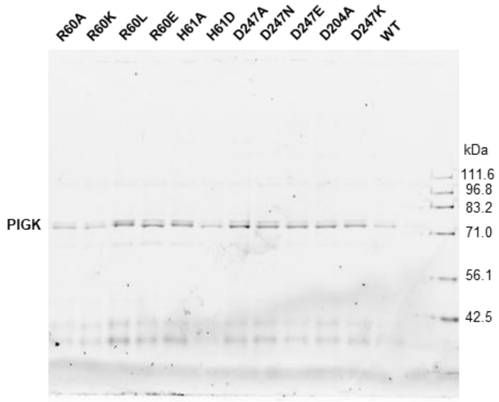

Related to Fig. 2b

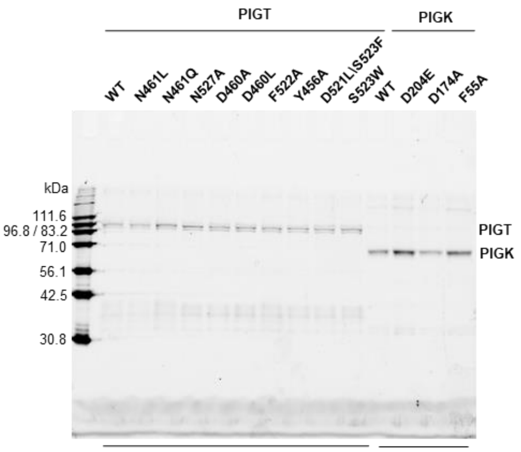

Related to Fig. 3d / Supplementary Fig. 6b

Related to Fig. 2b

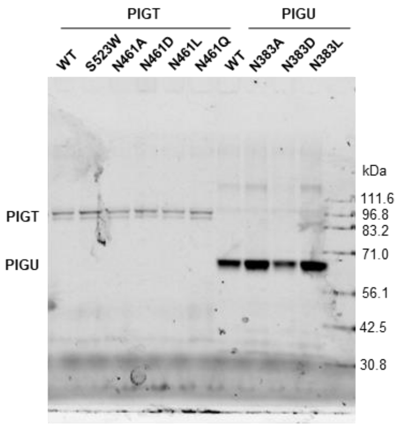

Related to Fig. 3d / Supplementary Fig. 6b

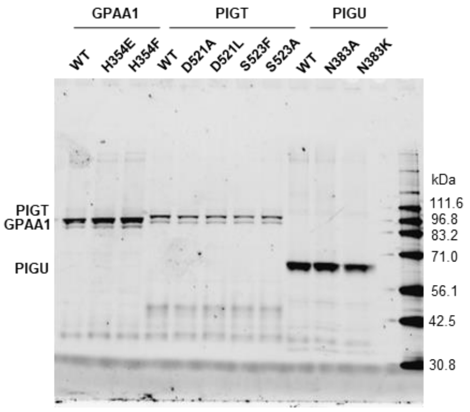

Related to Fig. 3d / Supplementary Fig. 6b

To be continued onto the next page

Continued from the previous page

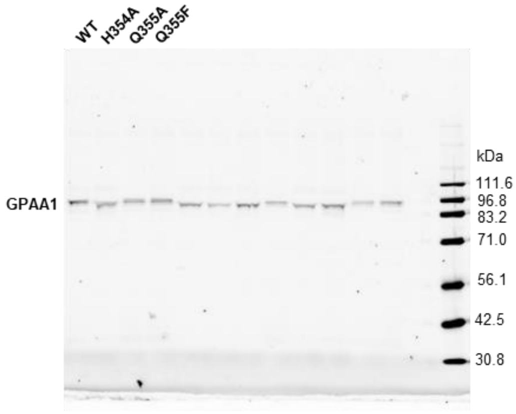

Related to Fig. 3d / Supplementary Fig. 6b

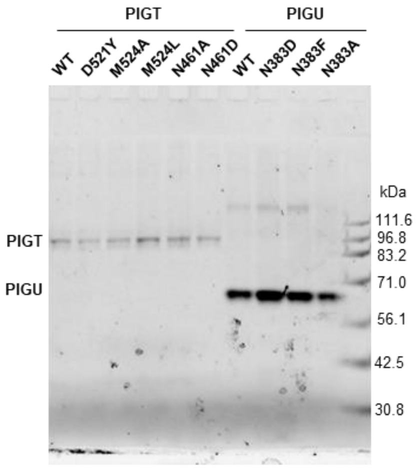

Related to Fig. 3d / Supplementary Fig. 6b

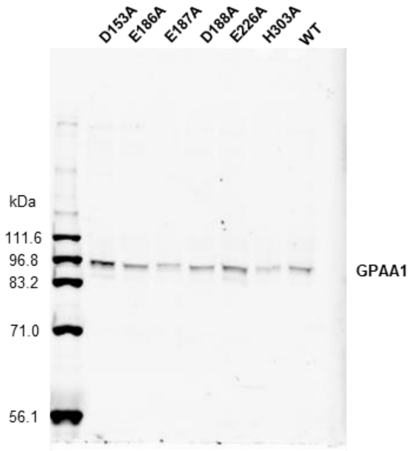

Related to Fig. 6e

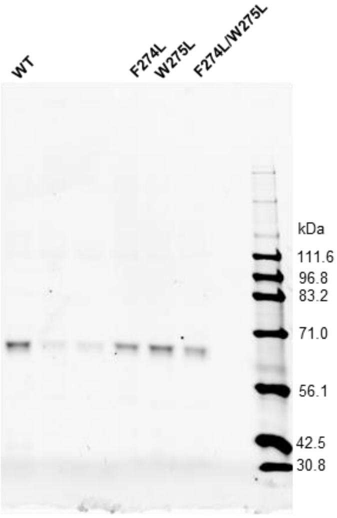

Related to Supplementary Fig. 9c

Supplementary Fig. 10. In-gel fluorescence of TGP-tagged subunits expressed in corresponding knockout (KO) cells suggest integrity of individual subunits. Theoretical Molecular weights of the homemade GFP markers [6] are indicated. Lanes are only labeled for the mutants reported in this paper. Low molecular-weight bands likely represent degradation products with an intact TGP tag. High molecular-weight bands may be glycosylated subunits or oligomers of intact or degraded subunits. The in-gel fluorescence was taken with a Fujifilm FLA-9000 gel scanner. Main figures and Supplementary figures reporting the mutants are indicated underneath each gel image.

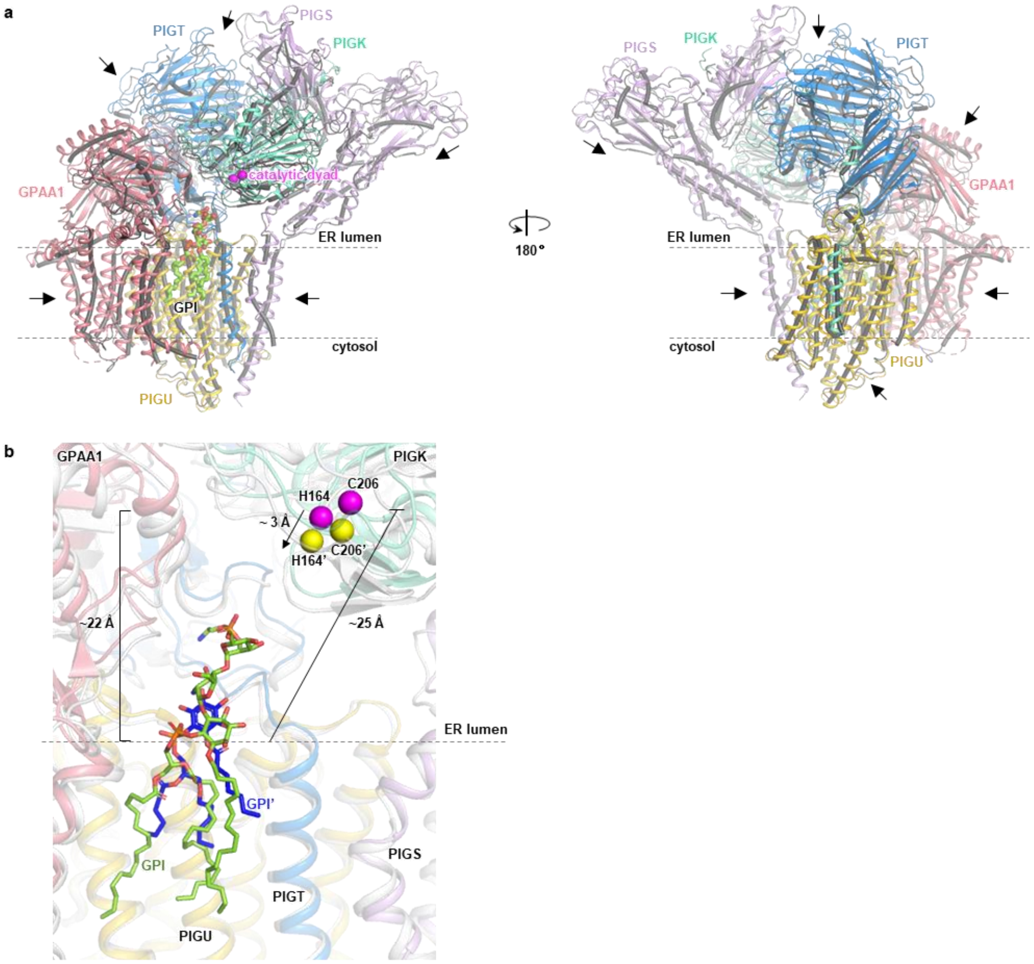

Supplementary Fig. 11. Comparison of GPI-T structures from Zhang *et al.* and this work. a Superposition of the recently published GPI-T structure [7] (3.10 Å, PDB ID 7W72 [http://doi.org/10.2210/pdb7W72/pdb]; grey, cylinder representation) with that determined in this work (2.53 Å, PDB ID 7WLD [http://doi.org/10.2210/pdb7WLD/pdb]; colored as indicated, cartoon representation). Arrows indicate local regions with position differences. The membrane boundary is marked by the dashed lines. **b** Expanded view of the active site of the two structures. Compared with the structure reported in this work, the catalytic dyad (H164, C206, magenta) in the 7W72 model (yellow) is ~3-Å closer toward GPI (7W72, blue; 7WLD, green). The protein part of the 7W72 model is colored light grey and its elements are labeled with a prime. The structure determined in this work is color-coded as in **a**. A dashed line indicates the membrane boundary at the endoplasmic reticulum (ER) lumen.

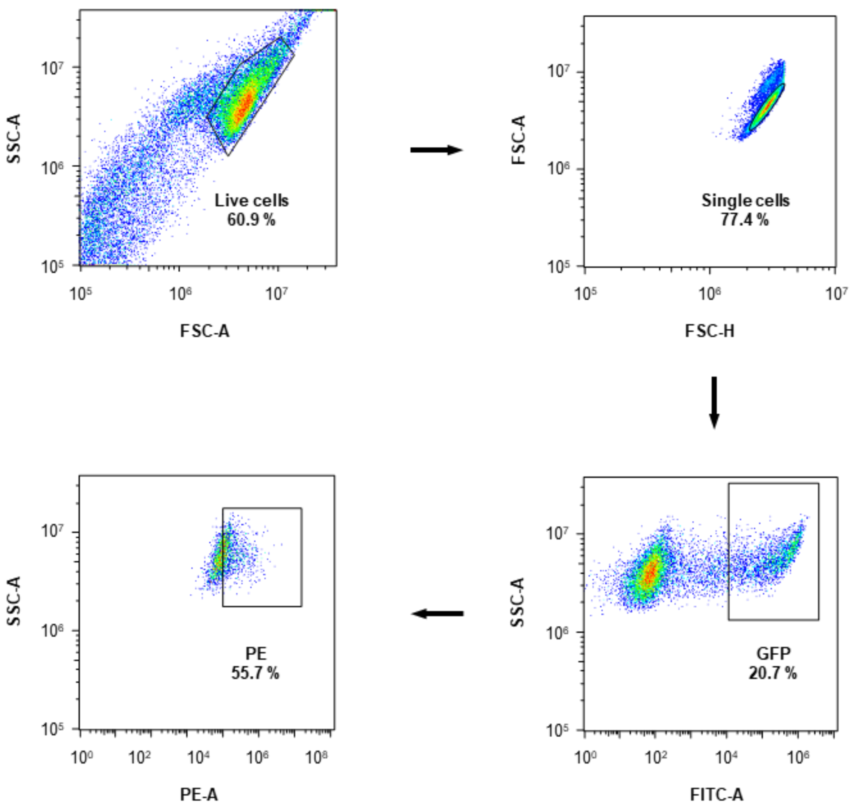

Supplementary Fig. 12. Representative flow cytometry gating strategy for the cell-based assay. GFP fused subunits (wild-type or mutants) were co-expressed in corresponding knockout cells. Apparent activity was assessed based on the surface staining of a GPI-AP reporter (CD59). The cells were first gated to select living cells (typically 50-60%) and single cells. The cells were then gated by GFP fluorescence to select the cells expressing TGP-fused subunits. The GFP positive subpopulation was then analyzed for the PE-positivity as an indication of the surface staining of the CD59. The data distribution is shown as a heat map with low counts in blue and high counts in red. FSC A/H, forward scatter area/height; FITC, fluorescein isothiocyanate (channel used in FACS analysis which detects GFP); GFP, green fluorescence protein; SSC A, side scatter area. PE, phycoerythrin (channel used for CD59 staining).

Supplementary Table 1. Cryo-EM data collection and refinement statistics.

	Human GPI-T complex
**Data collection and processing**	
Magnification	165,000
Voltage (kV)	300
Electron exposure ($e^-/Å^2$)	52.5
Defocus range (μm)	-2.4 to -0.6
Pixel size (Å)	0.85
Symmetry imposed	C1
Initial particle images (no.)	2,959,791
Final particle images (no.)	151,509
Map resolution (Å)	2.53
FSC threshold	0.143
Map resolution range (Å)	2.3 - 3.9
**Refinement**	
Model resolution (Å)	2.79
FSC threshold	0.5
Map sharpening *B* factor ($Å^2$)	77.5
Model composition	
Non-hydrogen atoms	19,733
Protein residues	2,393
Ligands	27
B factor ($Å^2$)	
Protein	50.8
Ligand	64.8
R.m.s. deviations	
Bond lengths (Å)	0.019
Bond angles (°)	1.325
Validation	
MolProbity score	1.80
Clashscore	10.66
Poor rotamers (%)	1.87
Ramachandran plot	
Favored (%)	97.77
Allowed (%)	2.15
Outliers (%)	0.08

References

1. Kinoshita, T. Biosynthesis and biology of mammalian GPI-anchored proteins. *Open Biol* **10**, 190290 (2020).
2. Dall, E. & Brandstetter, H. Mechanistic and structural studies on legumain explain its zymogenicity, distinct activation pathways, and regulation. *Proc Natl Acad Sci USA* **110**, 10940-10945 (2013).
3. Fuentes-Prior, P. & Salvesen, G.S. The protein structures that shape caspase activity, specificity, activation and inhibition. *Biochem J* **384**, 201-32 (2004).
4. Holm, L. & Rosenström, P. Dali server: conservation mapping in 3D. *Nucleic Acids Res* **38**, W545-W549 (2010).
5. Cai, H. et al. An improved fluorescent tag and its nanobodies for membrane protein expression, stability assay, and purification. *Commun Biol* **3**, 753 (2020).
6. Cai, H., Yao, H., Li, T., Tang, Y. & Li, D. High-level heterologous expression of the human transmembrane sterol Δ8,Δ7-isomerase in Pichia pastoris. *Protein Expr Purif* **164**, 105463 (2019).
7. Huang, X. et al. 11g, a Potent Antifungal Candidate, Enhances Candida albicans Immunogenicity by Unmasking β-Glucan in Fungal Cell Wall. *Frontiers in microbiology* **11**, 1324-1324 (2020).
