# Supplementary material for: Molecular insights into biogenesis of glycosylphosphatidylinositol anchor proteins
Source: Nat Commun. 2022 May 12;13:2617. doi: 10.1038/s41467-022-30250-6 (PMC9098846; doi:10.1038/s41467-022-30250-6)
Supplement: Supplementary file 3 — Reporting Summary [file 41467_2022_30250_MOESM3_ESM.pdf]

nature portfolio

Corresponding author(s): Zhaoming Su, Qianhui Qu , Dianfan Li

Last updated by author(s): Apr 16, 2022

Reporting Summary

Nature Portfolio wishes to improve the reproducibility of the work that we publish. This form provides structure for consistency and transparency in reporting. For further information on Nature Portfolio policies, see our Editorial Policies and the Editorial Policy Checklist.

Statistics

For all statistical analyses, confirm that the following items are present in the figure legend, table legend, main text, or Methods section.

n/a	Confirmed	
☐	☒	The exact sample size (*n*) for each experimental group/condition, given as a discrete number and unit of measurement
☐	☒	A statement on whether measurements were taken from distinct samples or whether the same sample was measured repeatedly
☒	☐	The statistical test(s) used AND whether they are one- or two-sided<br>*Only common tests should be described solely by name; describe more complex techniques in the Methods section.*
☒	☐	A description of all covariates tested
☒	☐	A description of any assumptions or corrections, such as tests of normality and adjustment for multiple comparisons
☐	☒	A full description of the statistical parameters including central tendency (e.g. means) or other basic estimates (e.g. regression coefficient) AND variation (e.g. standard deviation) or associated estimates of uncertainty (e.g. confidence intervals)
☒	☐	For null hypothesis testing, the test statistic (e.g. *F*, *t*, *r*) with confidence intervals, effect sizes, degrees of freedom and *P* value noted<br>*Give P values as exact values whenever suitable.*
☒	☐	For Bayesian analysis, information on the choice of priors and Markov chain Monte Carlo settings
☒	☐	For hierarchical and complex designs, identification of the appropriate level for tests and full reporting of outcomes
☒	☐	Estimates of effect sizes (e.g. Cohen's *d*, Pearson's *r*), indicating how they were calculated

Our web collection on statistics for biologists contains articles on many of the points above.

Software and code

Policy information about availability of computer code

Data collection	Preparative size exclusion chromatography, ChromLab 3.3.0.09; Cryo-EM data collection, EPU2 2.91; In-gel fluorescence, FLA-9000; Flow cytometry, Beckman CytoFlex LX, CytExpert 2.4.0.28, BD FACSAria Fusion, BD FACS Diva 8.0.3.
Data analysis	Cryo-EM data processing, Relion 3.1, MotionCor2, CTFFIND4 and CryoSPARC 3.1; 3D-model building, Coot 0.9.6; Structure refinement, Phenix 1.19.2-4158; Structure visualization, PyMOL 2.3.3 and ChimeraX1.1; FACS data analysis, FlowJo v10.0.7; Statistical analysis, GraphPad Prism 9.0.0.

For manuscripts utilizing custom algorithms or software that are central to the research but not yet described in published literature, software must be made available to editors and reviewers. We strongly encourage code deposition in a community repository (e.g. GitHub). See the Nature Portfolio guidelines for submitting code & software for further information.

Data

Policy information about availability of data

All manuscripts must include a data availability statement. This statement should provide the following information, where applicable:

- Accession codes, unique identifiers, or web links for publicly available datasets
- A description of any restrictions on data availability
- For clinical datasets or third party data, please ensure that the statement adheres to our policy

The coordinates for GPI-T model have been deposited in the PDB under accession code 7WLD [http://doi.org/10.2210/pdb7WLD/pdb]. The cryo-EM density map has been deposited in the Electron Microscopy Data Bank with accession code EMD-32582 [https://www.ebi.ac.uk/pdbe/entry/emdb/EMD-32582]. Uncropped images of Figs. 1b and 5d, and tabular data for Figs. 2b, 3d, 5e, 6e, and Supplementary Figs. 6b and 9e are provided as a Source Data file with this paper.

Field-specific reporting

Please select the one below that is the best fit for your research. If you are not sure, read the appropriate sections before making your selection.

[x] Life sciences [ ] Behavioural & social sciences [ ] Ecological, evolutionary & environmental sciences

For a reference copy of the document with all sections, see nature.com/documents/nr-reporting-summary-flat.pdf

Life sciences study design

All studies must disclose on these points even when the disclosure is negative.

Sample size	The sample size (n=3) is stated in the figure legends. Sample size was chosen based on previous experience and similar reports in the literature. No statistical methods were used to predetermine sample size.
Data exclusions	No data were excluded from the analysis.
Replication	All experimental results in this study are either from three independent experiments, or presented as a typical of at least three experiments. Similar results were obtained in repeated experiments using different cell batches and different plasmid preps and attempts to repeat the experiments were successful. Use of statistical methods have been described in relevant figure legends.
Randomization	The FACS assays sample a large number of cells that are from a single colony. Randomization was not relevant for this study.
Blinding	Because the data collection and analysis procedures were not subjective, there was no need for blinding.

Reporting for specific materials, systems and methods

We require information from authors about some types of materials, experimental systems and methods used in many studies. Here, indicate whether each material, system or method listed is relevant to your study. If you are not sure if a list item applies to your research, read the appropriate section before selecting a response.

Materials & experimental systems

n/a	Involved in the study	
[ ]	[x]	Antibodies
[ ]	[x]	Eukaryotic cell lines
[x]	[ ]	Palaeontology and archaeology
[x]	[ ]	Animals and other organisms
[x]	[ ]	Human research participants
[x]	[ ]	Clinical data
[x]	[ ]	Dual use research of concern

Methods

n/a	Involved in the study	
[x]	[ ]	ChIP-seq
[ ]	[x]	Flow cytometry
[x]	[ ]	MRI-based neuroimaging

Antibodies

Antibodies used	Phycoerythrin (PE)-labeled CD59 antibody (12-0596-42, clone OV9A2, Thermo Fisher Scientific, 1 : 500 dilution,)
Validation	The validation of the Phycoerythrin (PE)-labeled CD59 antibody is conducted by the manufacturer and the information can be found with the link: https://www.thermofisher.cn/order/genome-database/dataSheetPdf?producttype=antibody&productsubtype=antibody_primary&productId=12-0596-42&version=216

Eukaryotic cell lines

Policy information about cell lines

Cell line source(s)	HEK-293 cells, ATCC (Cat. CRL-3216); GPI-T knock-out cell lines, generated in this study; Expi-293 cells, ThermoFisher (Cat. A14527).
Authentication	The knock-out cell lines are verified using PCR, sequencing, and FACS analysis. HEK-293 cells and Expi-293 cells were not authenticated. Cells were maintained at lowest passage numbers possible to maintain identity.
Mycoplasma contamination	The cell lines were not tested for mycoplasma contamination.
Commonly misidentified lines (See ICLAC register)	No commonly misidentified cell lines were used in this study.

Flow Cytometry

Plots

Confirm that:

- [x] The axis labels state the marker and fluorochrome used (e.g. CD4-FITC).
- [x] The axis scales are clearly visible. Include numbers along axes only for bottom left plot of group (a 'group' is an analysis of identical markers).
- [x] All plots are contour plots with outliers or pseudocolor plots.
- [x] A numerical value for number of cells or percentage (with statistics) is provided.

Methodology

Sample preparation	Transfected wild-type or GPI-T KO HEK293 cells were washed with PBS, treated with trypsin as in the previous section, and washed and resuspended in 0.5 mL PBS. Phycoerythrin (PE)-labeled CD59 antibody (12-0596-42, Thermo Fisher Scientific, 1 : 500 dilution) was incubated with the cells for 15 min in dark. Cells were washed with PBS and resuspended in ~0.3 mL of PBS for flow cytometry (Beckman CytoFlex LX) monitored at two wavelength pairs (488/525 for GFP, 561/585 for PE).
Instrument	Beckman CytoFlex LX
Software	CytExpert 2.4.0.28 was used to collect the flow cytometry data. FlowJo v10.0.7 was used to analyze the flow cytometry data.
Cell population abundance	The commercial HEK cell lines has a cell population of 100%. The knockout cell lines we generated in this study was developed from single colonies and also have a cell population of 100%. Typically 40,000 cells were analyzed for each sample.
Gating strategy	Cells were gated using the GFP channel (from expression of TGP-tagged GPI-T subunit(s)) and analyzed for positive signal for the PE channel (for surface staining of CD59).

- [x] Tick this box to confirm that a figure exemplifying the gating strategy is provided in the Supplementary Information.
